# Optimizing of preoperative computed tomography for diagnosis in patients with peritoneal carcinomatosis

**DOI:** 10.1186/1477-7819-9-171

**Published:** 2011-12-21

**Authors:** Carolin D Duhr, Werner Kenn, Ralph Kickuth, Alexander G Kerscher, Christoph-Thomas Germer, Dietbert Hahn, Joerg O W Pelz

**Affiliations:** 1 Department of Radiology, University of Wuerzburg, Germany; 2 Department of General-, Visceral-, and Paediatric Surgery, University of Wuerzburg, Germany

**Keywords:** Carcinomatosis, PCI, diagnosis

## Abstract

**Background and Objective:**

This study evaluates whether Computer Tomography is an effective procedure for preoperative staging of patients with Peritoneal Carcinomatosis.

**Method:**

A sample of 37 patients was analyzed with contrast enhanced abdominal Computer Tomography, followed by surgical staging. All Computer Tomography scans were evaluated 3 times by 2 radiologists with one radiologist reviewing 2 times. The efficacy of Computer Tomography was evaluated using the Spearman correlation coefficient. Correlations were analyzed by abdominopelvic region to assess results of the Peritoneal Carcinomatosis Index (PCI) aggregating the 13 regions. Surgical findings were compared to radiological findings.

**Results:**

Results indicate high correlations between the surgical and radiological Peritoneal Carcinomatosis Indices. Analyses of the intra-class correlation between the first and second reading of one radiologist suggest high intra-observer reliability. Correlations by abdominopelvic region show higher values in the upper and middle regions and relatively lower values in the lower regions and the small bowel (correlation coefficients range between 0.418 and 0.726, p < 0.010; sensitivities range between 50% and 96%; and specificities range between 62% and 100%).

**Conclusion:**

Computer Tomography represents an effective procedure in the preoperative staging of patients with PC. However, results by abdominopelvic region show lower correlation, therefore suggest lower efficacy. These results are supported by analyses of sensitivity and accuracy by lesion size. This suggests that Computer Tomography is an effective procedure for pre-operative staging but less for determining a tumor's accurate extent.

## Introduction

Peritoneal Carcinomatosis ("PC") is a common metastasis location for many tumor variances with high incidences occurring in ovarian, gastric, and colorectal cancers. In literature, the occurrence frequencies for peritoneal metastases in ovarian, gastric, and colorectal cancers amount to 71%, 17%, and 10%, respectively [1,2]. Due to its natural history, PC is commonly associated with weak prognosis [3].

Today, various therapeutic procedures for PC exist with treatments being dependent on the PC's location and extent. The existence of peritoneal disease leads to different therapeutic procedures including exclusive use of systemic chemotherapy, cytoreductive surgery combined with or without hyperthermic intra-abdominal intra-peritoneal chemotherapy or exclusive palliative management. In order to achieve a highly selective group of patients a wide array of complex therapeutic procedures constitutes the current state of clinical research, including curative focused cytoreductive surgery ("CS") and hyperthermic intraperitoneal chemoperfusion ("HIPEC") [4-12].

Elias *et al. *demonstrated a median survival of 5 years with a 51% survival rate which is achieved by applying the HIPEC approach in a sample of well selected patients with PC of colorectal origin [13]. To ensure that patients benefit from multi-modality treatment, it is mandatory to apply this treatment on patients with limited peritoneal disease where a complete cytoreduction can be achieved. Contrast enhanced abdominopelvic computed tomography ("CT") is a frequently used pre-operative radiologic imaging modality to diagnose cancer within the abdominal cavity [3]. In former research the efficacy of CT diagnosing PC is not well established with an unknown size of implants being detected reliably [14]. Earlier work on CT of peritoneal malignancy was devoted to the detection of disease [15-19].

More recent studies analyzing the size of implants record varying sensitivities of 63% to 90% and specificities of up to 100% for the diagnosis of peritoneal metastasis [3]. However, in further work, sensitivities range from 41% to 79% and specificities are observed of up to 100% [20-22].

In a recent study by Coakley *et al. *ovarian carcinomatosis has been detected at relatively higher sensitivities of 85% to 93% [3]. Results were obtained with single-row CT scanners. The multi-row detector CT (MDCT) technology following the single-row CT scanner technology was able to show improved sensitivities and shortened examination time by allowing the generation of thin slices with subsequent multi-planar reconstruction (MPR). This technology was able to extents and metastatic spreads in patients with various malignancies as better imaging results were achieved through improved illustration of peritoneal implants. Calculated sensitivities are dependent on the lesion size ("LS") and are relatively weak for small lesion sizes; for example, sensitivities for lesions of less than 1 cm were observed between 20% and 25% resulting in low reliability of diagnosis quality [3,20,22].

A commonly applied regime for the quantification of the extent of PC and its deposits is the Peritoneal Cancer Index ("PCI") developed by Sugarbaker [23]. Today the PCI is acknowledged by research and used for determining therapeutic measures. It serves as an independent prognostic indicator for long-term outcomes in PC. The PCI is calculated as the sum of numerical lesion scores assigned to 13 abdominopelvic regions. The lesion score relates to the largest visible tumor deposit.

The objective of this study is to analyze the appropriateness and accuracy of CT as pre-operative diagnostic procedure and in combination with PCI as its indicator in detecting and determining the size and extent of PC.

## Materials and methods

Three individual readers of two radiologists and one surgeon evaluated pre-operative contrast enhanced abdominal CT scans for a population of 37 patients. Patients in the population underwent explorative laparotomy and were suspected to have PC from primary solid tumors.

Tumor spread, localization and size were described and documented applying Sugarbaker's PCI and lesion size schemes in both, radiological as well as surgical investigations. Radiological observations were retrospectively compared to surgical observations, whereby surgical findings were regarded as the Gold Standard. Radiological findings were then statistically analyzed using correlation analyses including inter-observer as well as intra-observer reliability analyses.

### CT protocol

All patients underwent CT scans according to a standardized CT acquisition protocol. Contrast enhancements were used including an oral contrast agent and the rectal filling Gastrolux (Sanochemia Diagnostics, Germany). All patients received intravenous injections of 110 mL Iomeprol (Imeron 300, Bracco Imaging, Germany) with a flow rate of 3 mL/s. A multi-slice CT scan (Siemens Somatotom Sensation 64, Germany) was used and all scans were conducted at 120 kV with 220 mAs as well as applying a care dose. Subsequent CT scans were started with a delay of 70 seconds. The collimation was 0.6 mm and the slice thickness was 5 mm, including coronal and sagittal reformations.

### Patient population

In this study a sample of 37 patients was investigated. Within this population 23 female and 14 male patients were between 24 and 78 years with an average age of 66 years at the time of the CT scan. All patients have shown solid primary tumors and underwent primary tumor surgery shortly after the CT scan (no more than 4 weeks).

In this population 32 patients had PC and 5 no PC; no patients were excluded from the study. The median time between CT scan and surgery was 10 days. Out of the 37 patients, 7 had colon cancer, 6 had gastric cancer, 5 had pseudomyxoma peritonei, 5 had ovarial cancer, 4 had pancreatic cancer, 3 had cancer of unknown primary, 2 had appendiceal cancer, and one patient each had adenocarcinoma of the abdominal wall, malignant mesenterial mesothelioma, gastrointestinal stomal tumor, mammary carcinoma, and pleural mesothelioma.

### System of tumor determination and description of PCI and LS score

**Figure 1:**
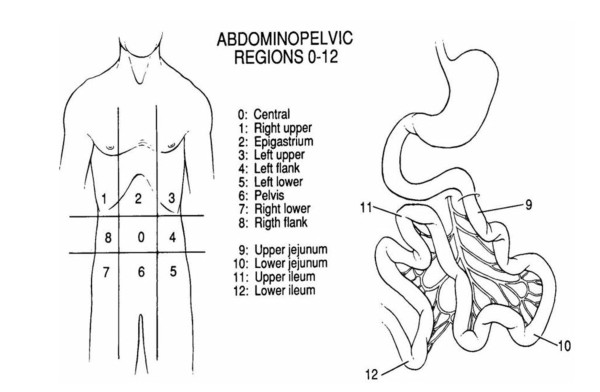
**The Sugarbaker's Peritoneal Cancer (PCI) describes the involvement of abdominal tumor mass**., [21].

The PCI is an accepted score for the quantification of tumor spread and localization. It serves as an indicator for therapy planning and prognosis. The entire abdominal and intestinal region is divided into 13 regions (Figure 1).

In each of the 13 regions the maximum visible lesion size is measured and assigned to a lesion size score between LS = 0 and LS = 3. LS = 0 means no tumor visible, LS = 1 means a tumor lesion size below 0.5 cm, LS = 2 means a tumor lesion size between 0.5 cm and 5 cm, and LS = 3 means a tumor lesion size larger than 5 cm or describes a confluent tumor.

LS scores in the individual regions are summed up to the PCI score which can assume a minimum score of 0 and a maximum score of 39.

The PCI is a semi-quantitative indicator for the determination of the extent of spread of peritoneal tumor. The success of complete cytoreductive surgery and prognosis of the patient correlates with the PCI. Patients with PC of colorectal origin with a reported PCI score of less than or equal to 10 have a 5 year survival rate of 50%; and lower survival rates of 20% and 0% for PCI scores between 11 and 20, and over 20, respectively.

### Radiological analyses

CT scans were conducted before explorative laparotomy with CT results being compared to intra-operative findings. In total, all CT scans were evaluated three times independently with some time-lag between each evaluation reading. Two radiological senior physicians with 20 years of experience evaluated the PCI scores independently from each other to achieve inter-observer reliability. Radiologist 1 reviewed scans twice to achieve intra-observer reliability, whereas Radiologist 2 only reviewed once. In order to obtain unbiased results evaluating radiologists were neither informed about the status of primary tumor nor the PC. In addition, a control group of 5 patients was added to the study that did not show PC in the explorative laparotomy.

### Surgical analyses

The explorative laparotomy and intra-operative data evaluations were conducted by a surgical team of one surgeon following a standard procedure and protocol. All patients underwent surgery in the same institution. Surgical and pathological findings were evaluated prospectively and documented.

In particular, the PCI score assignment was conducted by a surgical senior physician with 11 years experience in PC surgery. Intra-operative results regarding PC existence, lesion size, and localization served as Gold Standard.

### Statistical analyses

Radiological and surgical PCI scores were compared to each other applying the Spearman correlation coefficient to measure inter-rater reliability. Inter-observer reliability between two radiological readings was also analyzed using the Spearman correlation coefficient. Intra-observer reliability between the two readings of the same radiologist was measured using the intra-class correlation coefficient ("ICC"). All correlations were tested for statistical significance using the p-value. Different PCI scores were also evaluated for statistical difference applying the Wilcoxon rank test. In addition to correlation coefficients, both, sensitivities and specificities were calculated for the individual abdominal regions as well as lesion size. Statistical analyses were performed using Excel software with Analyse-IT statistical package.

## Results

### Differences in PCI results for intra-operative and radiological diagnosis

**Figure 2:**
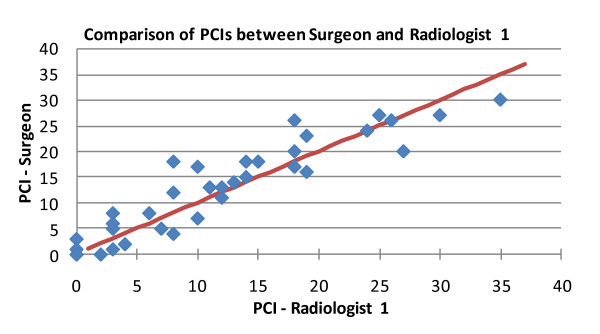
**Correlation 0.930 (p < 0.05) between radiological PCI and surgical PCI**.

PCI observations of 37 intra-operative procedures conducted by a single surgeon are compared with independent PCI observations of two radiologists. Key findings are high levels of correlation between the intra-operative and radiological observations showing correlation coefficients of around 0.9. In particular, compared to intra-operative findings, Radiologist 1 shows correlations of 0.930 (Figure 2) and 0.888 for his first and second reading, respectively. Radiologist 2 shows a correlation of 0.887.

All correlation coefficients in Table 1 is statistically significant at p-values below 0.001. In order to assess the reliability of findings, the *intra *and *inter-rater *reliabilities of observations by Radiologist 1 and Radiologist 2 were examined. Results for *intra-rater *reliability of Radiologist 1 first and second reading show high levels of reliability indicated by an intra-class correlation coefficient ("ICC") of 0.909 (p-value < 0.001). Correlation coefficients of 0.913 and 0.953 between readings of Radiologist 1 and Radiologist 2 demonstrate high *inter-rater *reliability.

**Table 1:** Overview of correlation coefficients.

	Radiologist 1		Radiologist 2
		
	1st Reading	2nd Reading	
Intra-operative	0,930	0,888	0,887
Intra-rater Reliability:			
1st Reading Radiologist 1		0,909	
	
Inter-rater Reliability:			
1st Reading Radiologist 1		0,953	
2nd Reading Radiologist 1		0,913	

Sensitivity and specificity between Radiologist 1 (first reading) and the Gold Standard are 94% and 80%, respectively (Table 2). Specificities are consistent for the other two radiological readings at 80% as both radiologists correctly detected 4 patients without PC (i.e., without an intra-operative PC finding) but observed 1 false positive for the same patient without PC. However, for this fifth patient without PC, PCI scores are PCI = 2 with 2 regions of LS = 1 for the first reading of Radiologist 1, PCI = 10 with 8 regions of LS = 1 and 1 region of LS = 2 for the second reading of Radiologist 1, as well as PCI = 7 with 7 regions of LS = 1 for the first reading of Radiologist 2. This demonstrates that the false positives were assigned small lesion sizes. The sensitivity for the second reading of Radiologist 1 is 94% corresponding to his first reading although true positives and/or false negatives are different by one patient. Radiologist 2 shows a sensitivity of 97% (Table 2).

**Table 2:** Overview of sensitivites and specificities of Radiologist 1 and Radiologist 2.

	Radiologist 1		Radiologist 2
		
	1st Reading	2nd Reading	
True positives	30	30	31
False negatives	2	2	1
Sensitivity	94%	94%	97%
True negatives	4	4	4
False positives	1	1	1
**Specificity**	**80%**	**80%**	**80%**
			
**PPV**	**97%**	**97%**	**97%**
**NPV**	**67%**	**67%**	**80%**

In most relations, the Wilcoxon rank test, used to analyze differences among the intra-operative observations and radiologist observations, indicates statistically insignificant differences at p-values > 0.05 (Table 3).

**Table 3:** Significance of difference in results demonstrated with the Wilcoxon rank test (p-Value).

Significance of difference in results			
	First Reading	Second Reading	Reading
Wilcoxon rank test (p-Value)	Radiologist 1	Radiologist 1	Radiologist 2
Intra-operative (surgeon)	0,35	0,42	0,02
First Reading Radiologist 1		0,66	0,02
Second Reading Radiologist 1			0,08

In light of high levels of correlation between intra-operative and radiological observations the insignificantly different recorded findings suggest that both radiologists evaluated the magnitude of PCI insignificantly differently than the surgeon (i.e. Gold Standard).

### CT analysis by abdominopelvic regions

Correlation analyses by abdominopelvic region were conducted between the intra-operative findings (Gold Standard) and the first reading of Radiologist 1. Results by region show varying correlation coefficients ranging from 0.418 to 0.881 at statistically significant levels (p-value < 0.05) (Table 4).

**Table 4:** Results by abdominopelvic region including correlation coefficient, p-value, sensitivity, specificity, underestimation, and overestimation.

	Abdominopelvic region												
	
	Upper			Middle			Lower			Small Bowel			
	**1**	**2**	**3**	**8**	**0**	**4**	**7**	**6**	**5**	**9**	**10**	**11**	**12**
Correlation	0,748	0,713	0,660	0,823	0,774	0,881	0,659	0,726	0,698	0,418	0,431	0,519	0,533
*P-value*	*0,000*	*0,000*	*0,000*	*0,000*	*0,000*	*0,000*	*0,000*	*0,000*	*0,000*	*0,010*	*0,008*	*0,001*	*0,001*
													
Sensitivity	86%	86%	91%	96%	86%	100%	96%	88%	93%	50%	53%	70%	70%
Specificity	87%	75%	73%	79%	75%	94%	62%	69%	80%	89%	94%	100%	100%

Under estimated	14%	14%	9%	4%	14%	0%	4%	13%	7%	50%	47%	30%	30%
Over estimated	13%	25%	27%	21%	25%	6%	38%	31%	20%	11%	6%	0%	0%

**Figure 3:**
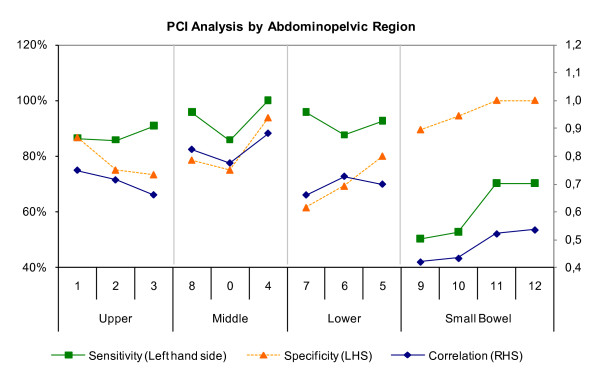
**Analyses of abdominopelvic regions including correlation, sensitivity and specificity**.

The analysis of results shows relatively higher correlations in the upper and middle abdominopelvic regions compared to the lower region and the smaller bowel. However, the upper and middle abdominopelvic regions show lower sensitivity and specificity at 86% and 73%, respectively, compared to the lower abdominopelvic region. This result suggests a relatively high CT performance. CT performance in the smaller bowel is inferior with lower levels of correlation and levels of sensitivity below 0.6 and 70%, respectively (Figure 3).

CT performance is high in the middle abdominopelvic regions which have more evenly distributed lesions sizes of 1, 2, and 3 compared to the upper region with a concentration around lesion size 1 (Table 5).

**Table 5:** Distribution of lesion sizes in the various abdominopelvic regions.

	Abdominopelvic region												
	
	Upper			Middle			Lower			Small Bowel			
Lesion size	1	2	3	8	0	4	7	6	5	9	10	11	12
LS = 0	15	16	15	14	16	16	13	13	10	19	18	17	17
LS = 1	11	12	14	8	5	10	9	10	15	14	16	18	17
LS = 2	6	4	4	8	7	7	11	11	7	3	2	1	2
LS = 3	5	5	4	7	9	4	4	3	5	1	1	1	1
													
LS = 0	**41%**	**43%**	**41%**	**38%**	**43%**	**43%**	**35%**	**35%**	27%	**51%**	**49%**	46%	**46%**
LS = 1	30%	32%	38%	22%	14%	27%	24%	27%	**41%**	38%	43%	**49%**	**46%**
LS = 2	16%	11%	11%	22%	19%	19%	30%	30%	19%	8%	5%	3%	5%
LS = 3	14%	14%	11%	19%	24%	11%	11%	8%	14%	3%	3%	3%	3%

Differences in results by abdominopelvic region appear to be due to different lesion sizes (excepted for small bowel evaluation) and not because of anatomic or pathologic differences.

### Analysis by lesion size

Analysis of findings by lesion size show that results depend on lesion size and that sensitivities are improved with increasing lesion sizes but matching observations (defined as the number of correct radiological tumor size observations divided through the number of total observations) show a declining rate (Table 6).

**Table 6:** Analysis of findings by lesion size demonstrating true positives, false negatives, sensitivity, specificity, accuracy, underestimation, overestimation.

	LS = 0	LS = 1	LS = 2	LS = 3
Region count	199	159	73	50
True positives	n.a.	118	71	45
False negatives	n.a.	41	2	5

Sensitivity	n.a.	74%	97%	90%
Specificity	84%	n.a.	n.a.	n.a.
Accuracy	84%	53%	48%	34%

Under estimate	n.a.	26%	34%	66%
Over estimate	16%	21%	18%	n.a.

n = 481 (37 patients × 13 abdominopelvic regions)

n.a. = not available

While sensitivities increase from 74% for LS = 1 to 90% for LS = 3 with a peak at LS = 2 of 97%, the proportion of actually matching observations between the Gold Standard and Radiologist 1 declines from 84% for LS = 0 to 34% for LS = 3.

## Discussion

PC is a metastasis derived from different primary tumors such as the ovarian, the abdominal, and the colorectal carcinomas (in order of highest frequency). In many cases, a progressed stadium of tumor can be observed in connection with diagnosed PC and is often associated with a negative prognosis. The survival rate of patients with peritoneal metastases from colorectal origin is low with 5 to 32 months [24].

Several treatment methods exist for the therapy of PC. A multimodal treatment procedure introduced in 1990 is commonly used including cytoreductive surgery with subsequent intra-abdominal and intra-peritoneal hypothermic chemotherapy. This treatment procedure allows a 5 year survival rate of 30% to 45% [25].

The outcomes of PC diagnosis and prognosis are primarily dependent on the parameters of tumor spread, localization, and lesion size. Exact determinations of these parameters is therefore of clinical importance for determining and improving common prognosis and therapy planning. In clinical practice it is of importance to decide which therapy regime to choose, and it is furthermore important to find out if a patient has to be excluded from a certain therapy regime or not. Patients with a PCI over 20 are regarded not to be appropriate for cytoreductive surgery ("CRS").

Most studies were conducted with ovarian carcinoma with primary tumor. Further studies with patients with PC of non-gynecological origin such as the colorectal carcinoma would be of clinical interest.

In our study the correlation between radiological and intra-operative finding is very high with a correlation coefficient around 0.9. All correlations were statistically significant at p-values below 0.001.

Notable are correlation coefficients of single regions that are below 0.900 and range between 0.418 and 0.881 compared to an overall PCI correlation of 0.930. This suggests that the PCI, as summation of the observations in the 13 regions, could be misleading due to its aggregating effect. Through this summation, potential individual differences in observations could compensate each other leading to an overall lower PCI difference.

The distribution of lesion sizes in the various abdominopelvic regions is shown in Table 5.

Evaluations of the PCI Scores are depending on the expertise and experience of the radiologist and surgeon. For this reason another independent reading of the CT scans by a second radiologist was included in this study. We measured a high inter rater reliability between both radiologists of 0.953 and 0.913 at p values below 0.001.

Furthermore, to assess the reliability of findings one radiologist evaluated CT scans a second time. The intra-rater reliability supported by in the intra class coefficient amounted to 0.909 (p < 0.001) which could be considered very high.

This study shows that both radiologists with a similar background of experience evaluated CT scans with high levels of correlation. These finding are in line with other recent published studies.

In a study by Coakley *et al. *in which preoperative CT scans of patients with ovarian carcinoma were reviewed by three independent readers, showed that the depiction of peritoneal metastases is good to excellent [3]. The Kappa value in his study ranged between 0.75 to 0.91 which indicates good to excellent inter-observer agreements.

This study shows an overall sensitivity of 94% and specificity of 80% which are broadly in line with more recent studies. Studies by Coakley *et al. *and Tempany *et al. *showed sensitivities of 85% to 93% in the detection of peritoneal metastases in patients with ovarian cancer [3,26].

Correlation was analyzed by abdominopelvic regions ranging from 0.418 to 0.881 at statistically significant levels. Higher correlations were found in upper and middle abdominopelvic regions compared to lower region and small bowel. Upper and middle abdominopelvic regions show higher sensitivities and specificities above 86% and 73%, respectively, compared to the lower region.

The best evaluated regions were left flank (4) with 100% sensitivity, right lower (7) and right flank (8) with 96% and left lower (5) with 93%. The inclusion of the smaller bowel plays an important part in the prognosis and a reason for including it in this study regarding tumor extent and size. In line with finding in previous studies, CT is less appropriate for diagnosing the small bowel region. In this region, results show the lowest sensitivities in this study of 50% to 70% and correlation levels between 0.418 and 0.533. The study by Koh *et al. *shows sensitivities of 8% to 14% in the small bowel region which can also be found in a study by de Bree *et al. *[4,14]. The improvement of sensitivities in this study is expected to be a result of the application of a single center setup with a highly standardized CT acquisition protocol. Furthermore CT scans in this study were performed with oral and intravenous fillings and reconstruction. It seems probable that a standardized CT acquisition protocol leads to better results in detection rates. A possible reason for low sensitivity in the detection of PC diseases could be the time lag between the CT scan and the operative surgery during which period a tumor can potentially grow. These results observations are supported by high correlations between radiological and surgical findings and high levels of inter and intra-rater reliability.

The relatively lower performance of CT in the upper region can be explained by a concentration of lesions sizes of 1 where CT shows a low sensitivity. Low sensitivity and high specificity in the small bowel could stem from a high concentration of lesion sizes of 1 and 0, respectively.

It is shown that the PCI score is depending on lesion size. With increasing lesion size the sensitivity is improving; however, accurate observations are declining with increasing lesion size. Sensitivity increases from 74% for LS = 1 to 90% for LS = 3, while the accuracy between surgical and radiological observations is declining from 84% for LS = 1 to 34% for LS = 3. A declining accuracy with an increasing lesion size is explained by increasing underestimation by the radiologist. It is observed that the radiologist systematically underestimates the Gold Standard which is supported by the findings according to lesion size.

There exist several methods for imaging PC including CT, magnet resonance tomography, FDG-PET-Observation, or ultrasound. The most used and commonly applied method is the CT based diagnosis. Subject of this study is the assessment of appropriateness and accuracy of CT for the detection and diagnosis of PC.

Due to comparatively higher availability, lower costs, and evaluation artifacts of bowel peristaltic and breathing, CT with oral or intravenous contrast injection is still the most commonly used method for the diagnosis of PC [27].

In studies from 1985 until 2009 evaluating the appropriateness of CT for the detection of PC, sensitivities reach from 14% to 93% and specificities from 54% to 100%. Such spectrums are expected to be the result of the advent of new technology including the multi-detector-CT or the multimodal reconstruction. The recent study by Coakley *et al. *showed sensitivities of 85% to 93% and specificities of 91% to 96% for CT based PC diagnosis [3].

The increasing improvement of results could be due to the further development of technology such as the contrast enhanced spiral CT. In a study by Jacquet *et al. *sensitivities were first described in relation to tumor lesion size; sensitivities were recorded at 70% for a maximum lesion size of 2 cm whereas at 28% for lesion sizes of less than 0.5 cm [22].

Coakley *et al. *investigated sensitivities in relation to lesion size and showed 25% to 50% lower sensitivities for lesion sizes below 1 cm compared to overall observed sensitivities between 85% and 93%, thereby suggesting lower performance of CT for small lesion sizes [3]. Further studies investigating CT performance for small lesion sizes would be of clinical interest.

The study by Tempany *et al. *investigated sensitivities in relation to origin of primary tumor; sensitivity of 92% was observed with patients with ovarian carcinoma [26]. Similarly, the study by de Bree *et al. *showed a sensitivity of 60% to 76% for patients with appendenix and colorectal carcinoma [20]. In most studies sensitivities were below 20% for the regions of mesenterium and small bowel [14]. According to de Bree *et al. *the surface can best be observed with the existence of ascites [20]. In this study the existence of ascites did not explain significantly better results.

With the introduction of more advanced technologies imaging quality has been improved. Coronary and sagittal imaging methods result in the detection of smaller tumor lesion sizes with smaller artifacts [28]. With the advent of higher resolutions of scanner improved depiction of PC could expected.

A disadvantage of CT is the distinguishing of the tumor scar tissue from the post-operative scar tissue which is almost impossible with current sophistication of CT technology.

A study by Franiel *et al. *investigated the impact of thinner layers and multi-planar reconstruction in connection with the observation of PC [29]. Layers of 5 mm thickness were found to be sufficient whereas layers of 1 mm in connection with MPR could improve sensitivities and diagnostic confidence. There, results were dependent on the radiologist's years of experience. A radiologist with over 10 years of experience, using 0 coronal and sagittal MPRs, could achieve an improved sensitivity of 96% when using 1 mm thin slices compared to 86% when using 0.5 cm thin slices. Best results with sensitivities of up to 100% could be shown by using MPRs.

MRI and spiral CT show similar accuracy in the detection of PC and PC size, spread and localization compared to CT. In a study by Kim *et al. *it is showed that MRI achieves a sensitivity of 95% [30]. The mandatory long hold of breath proves to be difficult for many patients with tumors due to their generally weak condition. This could lead to evaluation artifacts and diminish the quality of MRI depiction [30]. Another disadvantage is the existence of ascites which could also lead to artifacts using MRI and could often be the reason for high wrong positive-rates [30].

Tempany *et al. *in his study investigated MRI in comparison to CT wherein higher sensitivities for MRI of 95% compared to 92% for CT could be shown [26]. However, specificities were lower at 80% for MRI compared to 82% for CT. Overall the accuracy was not significantly different between the two modalities. Although MRI performed better at sensitivities between 85% to 90% for small modular peritoneal changes of less than 1 cm compared to CT with 25% to 50% it could questionable whether MRI is a superior imaging method given its relatively longer duration of observation and lower availability. In previous literature, the sensitivity of MRI for the detection of PC is approximately 85% [31,32] and specificity of around 80% [26].

The appropriateness and effectiveness of PET-CT for the diagnosis of PC has yet to be investigated. The study by Dromain *et al. *concludes that neither CT nor PET-CT could be used stand-alone for the prognosis and planning of PC therapy due to a correct detection evaluation of 70% for CT and 80% for PET-CT [33].

The FDG-PET (2-Fluor-2-deoxy-D-Glucose positron emission tomography) is another alternative method but showed low specificities between 54% and 86% [34,35]. FDG-PET also shows lower accuracy for the detection of smaller lesion sizes compared to MRT und CT [36].

The laparotomy is still the preferred method for the exact staging of investigation with histological biopsy. It is, however, also associated with peri-operative dissemination of maligned cells. Furthermore, the invasive procedure is often associated with higher rates of mortality and morbidity [37-39].

Laparoscopy is also an effective method for staging peritoneal carcinomatosis. Unfortunately, not every region can be assessed. Therefore, laparoscopy appears to be a highly suitable method of diagnosis for the small bowel in cases of vague involvement.

In line with previous research, this study underpins that CT is an effective imaging modality for the pre and postoperative staging of patients. A standardized CT acquisition protocol in a certified PC center and readings performed by highly experienced radiologists could result in sufficiently high sensitivity and specificity rates for an effective patient selection process. Relatively high sensitivities and specificities results are achieved by abdominopelvic region as well as by lesion size. However, the appropriateness of CT for the evaluation of the small bowel region is still insufficient and needs to be further investigated.

## Conclusion

A standardized CT acquisition protocol in a certified peritoneal cancer center and readings performed by highly experienced radiologists seem to achieve higher values for sensitivity and specificity and therefore are expected to result in a more effective patient selection process.

## Competing interests

The authors declare that they have no competing interests.

## Authors' contributions

CCD participated in the design, acquisition, analysis and interpretation of data, drafting manuscript, critical revision. WK carried out the first radiologist reading. RK carried out the second radiologist reading. AK participated in drafting and editing of manuscript, data analysis. CTG participated in the acquisition of data, critical revision. DH carried out in the data analysis and Critical revision. JOWP participated in the design, acquisition, analysis and interpretation of data, drafting manuscript, critical revision. All the authors participated in the conception, the design, data collection and interpretation, manuscript preparation, and literature search. All authors have read and approved the final manuscript.
